# Supplementary material for: Hierarchical modularity of nested bow-ties in metabolic networks
Source: BMC Bioinformatics. 2006 Aug 18;7:386. doi: 10.1186/1471-2105-7-386 (PMC1560398; doi:10.1186/1471-2105-7-386)
Supplement: Additional File 1 — Supplementary material for this paper [file 1471-2105-7-386-S1.pdf]

Supplementary for “Hierarchical modularity of nested bow-ties in metabolic networks”

I. Bow-tie topological feature and modules of the *E.coli* metabolic network

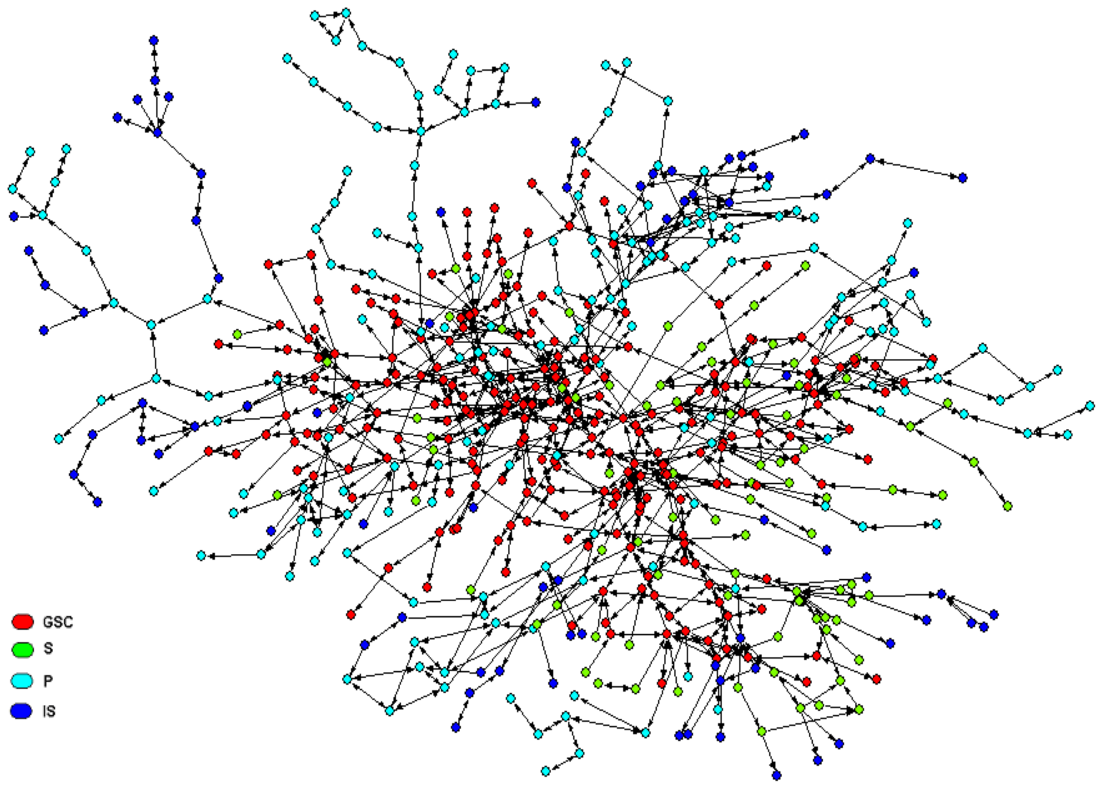

Figure S1 Bow tie structure of E.coli network. The GSC, S, P and IS part include 234, 85, 177, 79 nodes respectively.

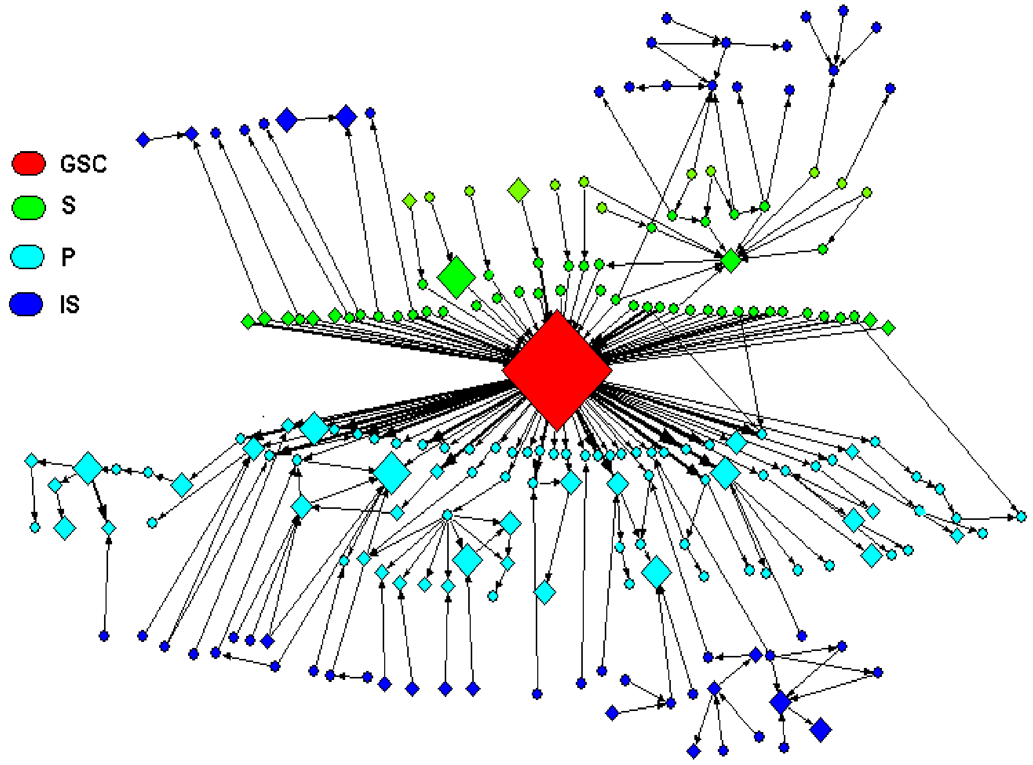

Figure S2 Bow-tie structure in the coarse-grained graph of the E.coli metabolic network, in which every strongly connected component has been shrunk into a diamond node. A bigger diamond corresponds to a strong component with more nodes, while a thicker arc represents more links between the corresponding clusters. The biggest strongly connected component (GSC) includes 234 nodes, while the second biggest one has only 11 nodes. As illustrated in the paper, nodes in the same strongly connected component are structural equivalent. Therefore, this coarse-grained graph is a kind of equivalent reduction of the metabolic network, which keeps its metabolic flows. In this way, the original interleaving and complicated metabolic network has been summarized into a clearly branched bow-tie model, which helps to illustrate the global biological metabolic flow.

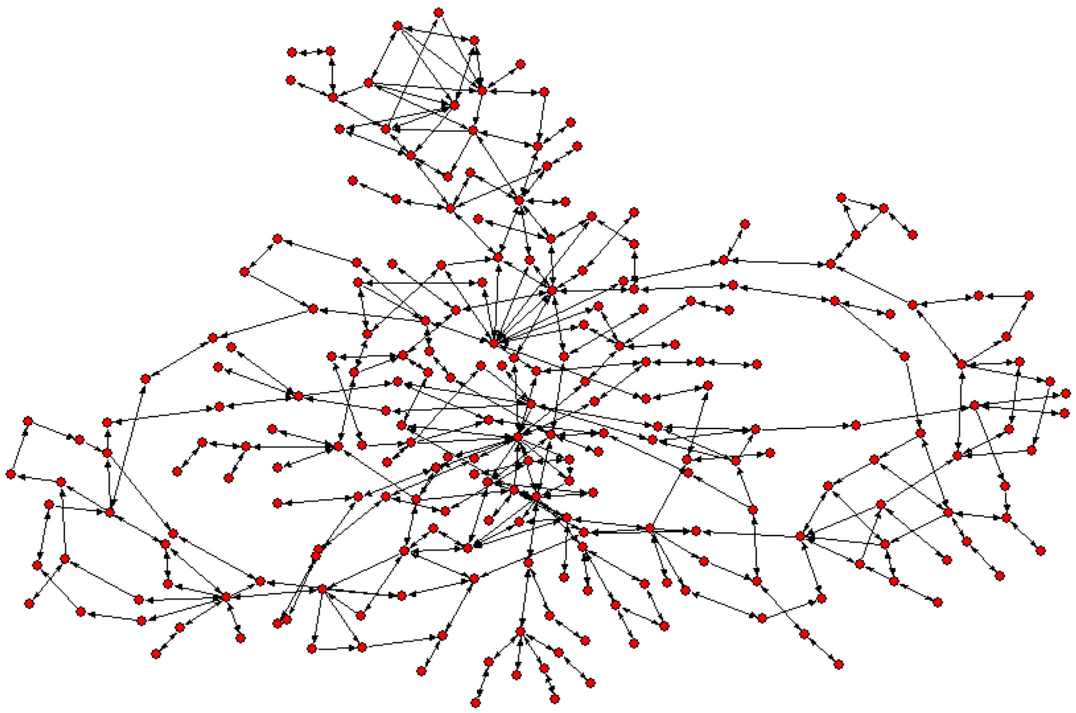

Figure S3 GSC part of the E.coli network. It includes 234 nodes.

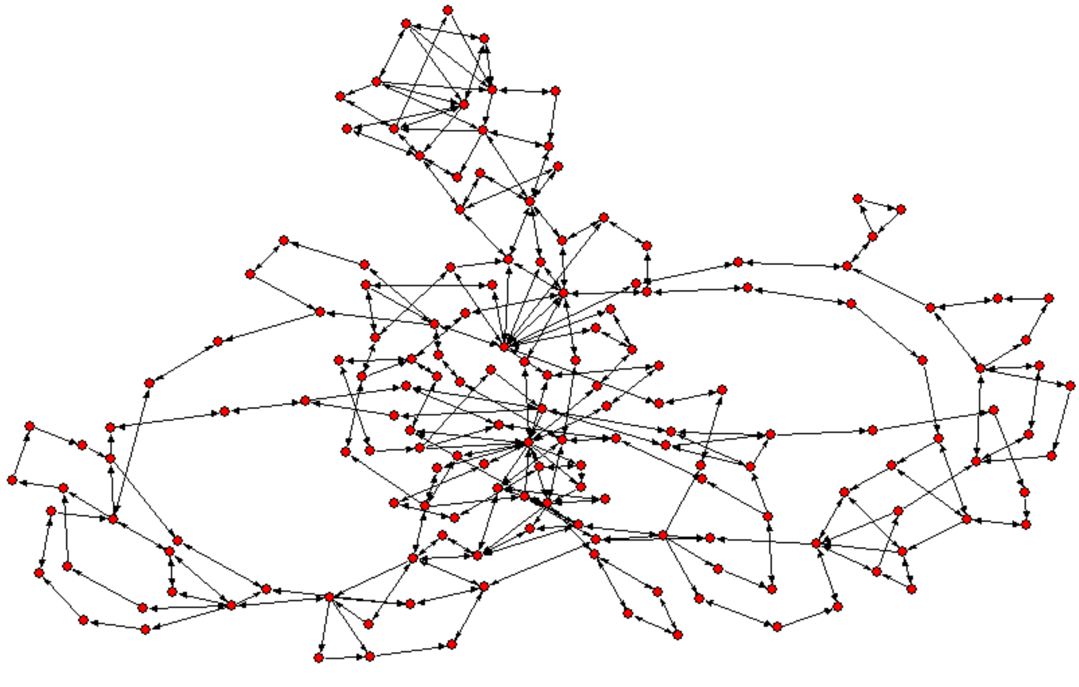

Figure S4 Core of the GSC part for the E.coli network, obtained by removing all the linear branches of GSC. It includes 163 nodes.

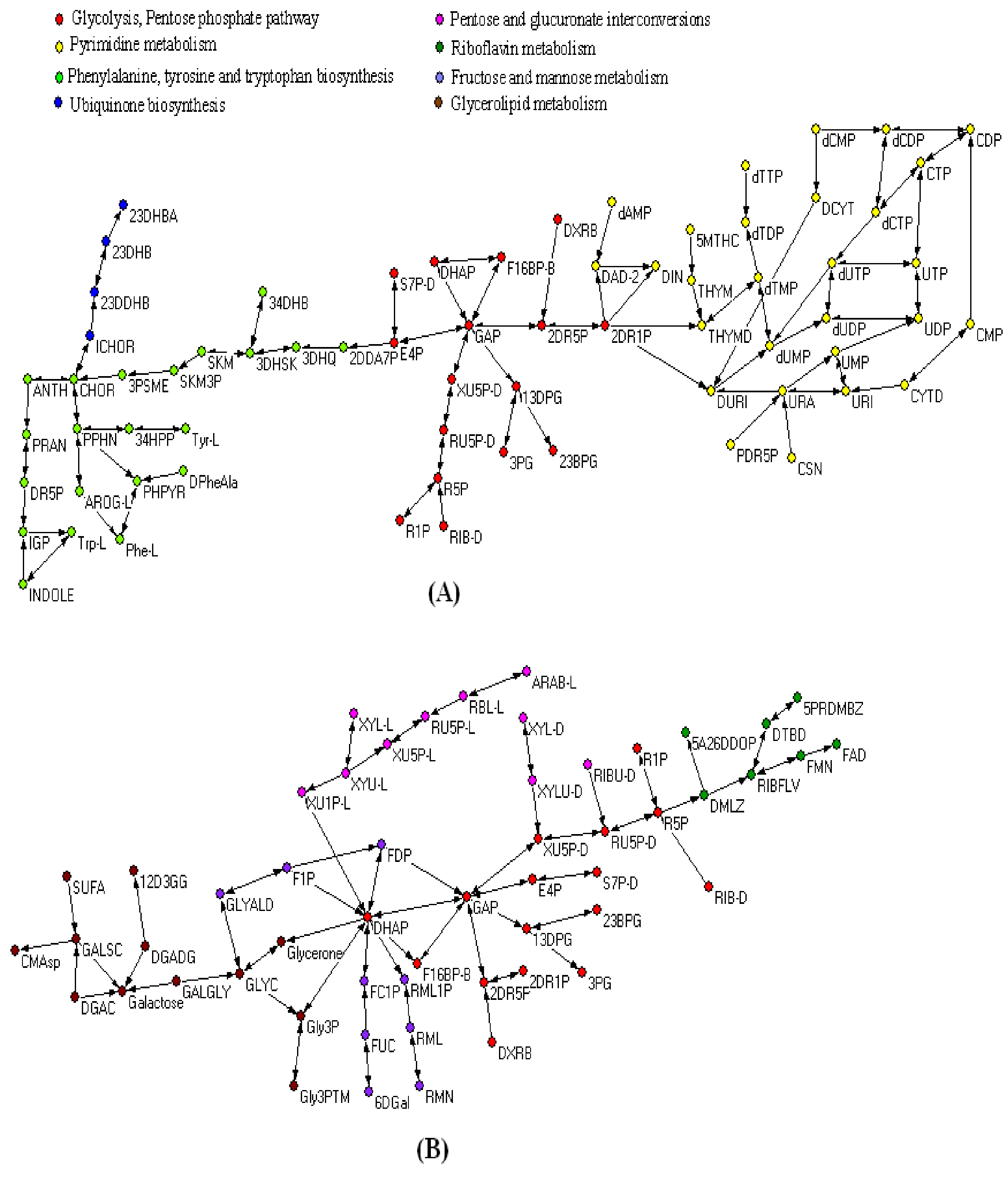

Figure S5 Pathways in the 5th module

(A) and (B) show different parts of module 5 that are linked by glycolysis and pentose phosphate pathway. It can be seen that glycolysis and pentose phosphate pathway act as “pivot” of the 5th module. The metabolite abbreviations are listed in Additional file 2.

II. Breaking up the network for *Aeropyrum pernix*(ape) as an illustration of the decomposition algorithm: detail steps

1. Remove all the linear branches of the GSC part and get the Core

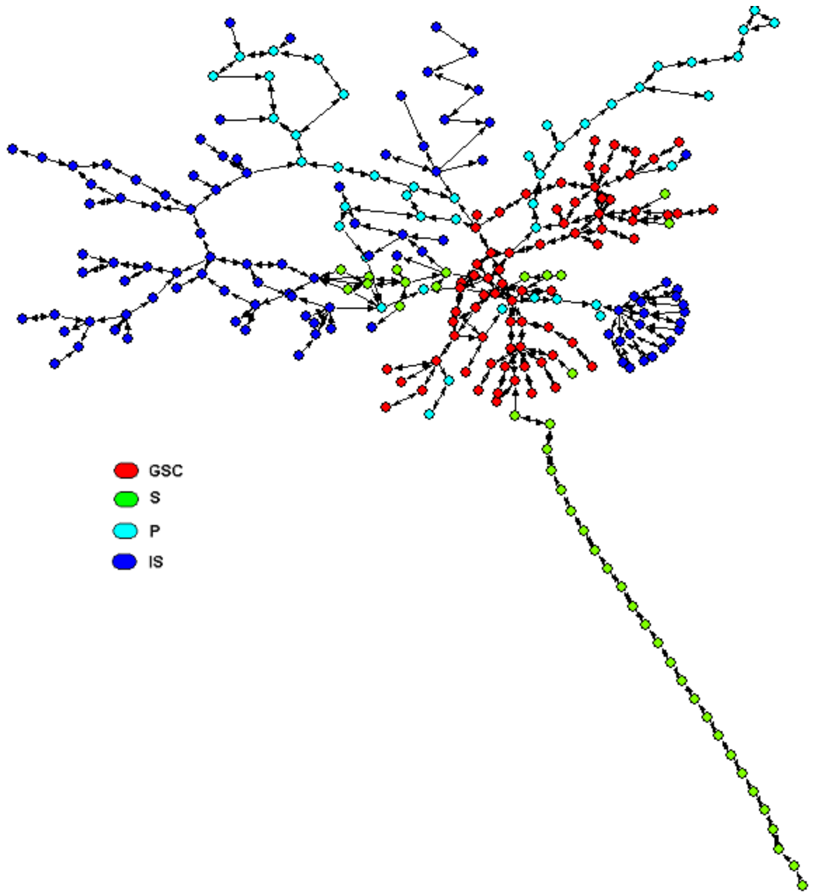

Figure S6 Bow tie structure of ape network. The GSC, S, P and IS part include 73,41,45,97 nodes respectively.

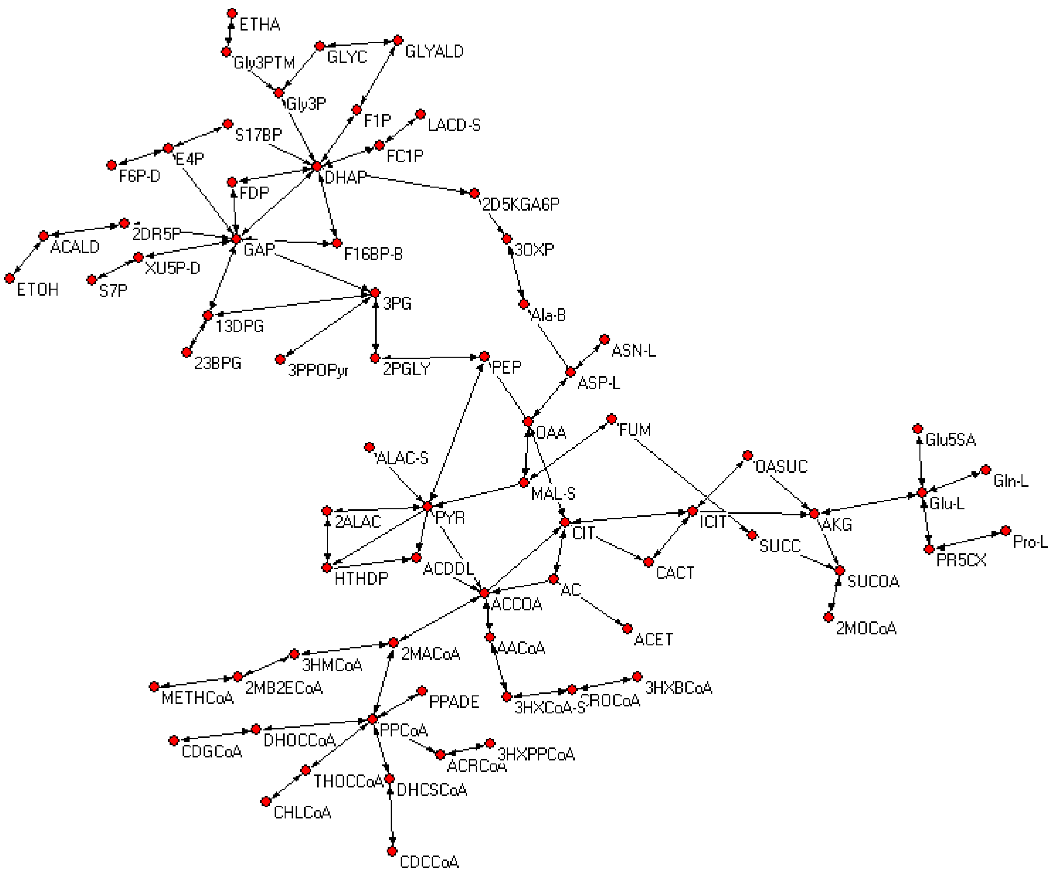

Figure S7 GSC part of the ape network. It includes 73 nodes. The metabolite abbreviations are listed in Additional file 3.

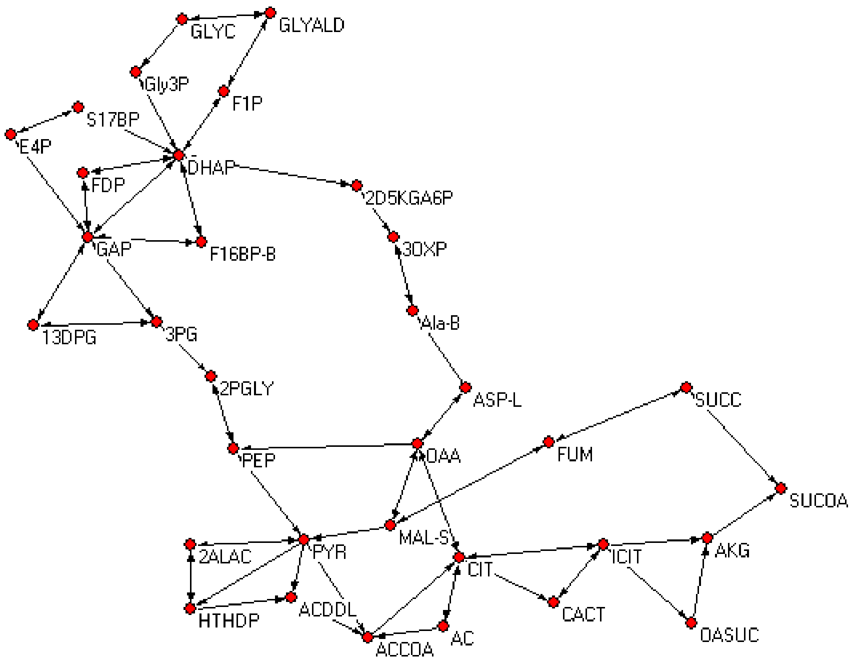

Figure S8 Core of the GSC part for the ape network, obtained by removing all the linear branches of GSC. It includes 34 nodes.

2. Decompose the Core of the GSC by Ward's clustering and get its hierarchical clustering tree, and then cut the hierarchical clustering tree into 4 clusters so that every cluster corresponds to a connected sub-graph, while the value of modularity metric is the largest.

Figure S9 and Figure S10 compare the decomposition results corresponding to cutting the hierarchical clustering tree into 4 and 5 sub-trees. It is worth to note that with the dissimilarity index of equation (1), nodes that belong to the same sub-tree own the highest degree of "structural equivalence", but are not necessarily connected to each other. For example, Figure S10(B) shows that the 5 nodes corresponding to the 5th sub-tree in Figure S9(B) are not linked together, i.e., the decomposition of Figure S9(B) generates clusters smaller than 4, thus it is not accepted by our algorithm (we set the threshold of the smallest cluster as 4 nodes for statistical significance).

Figure S10 also shows that the Core part has been decomposed by our algorithm in such a way that the clusters are still strongly connected (cluster 1 and 4), or most of the nodes are strongly connected (cluster 2 and 3).

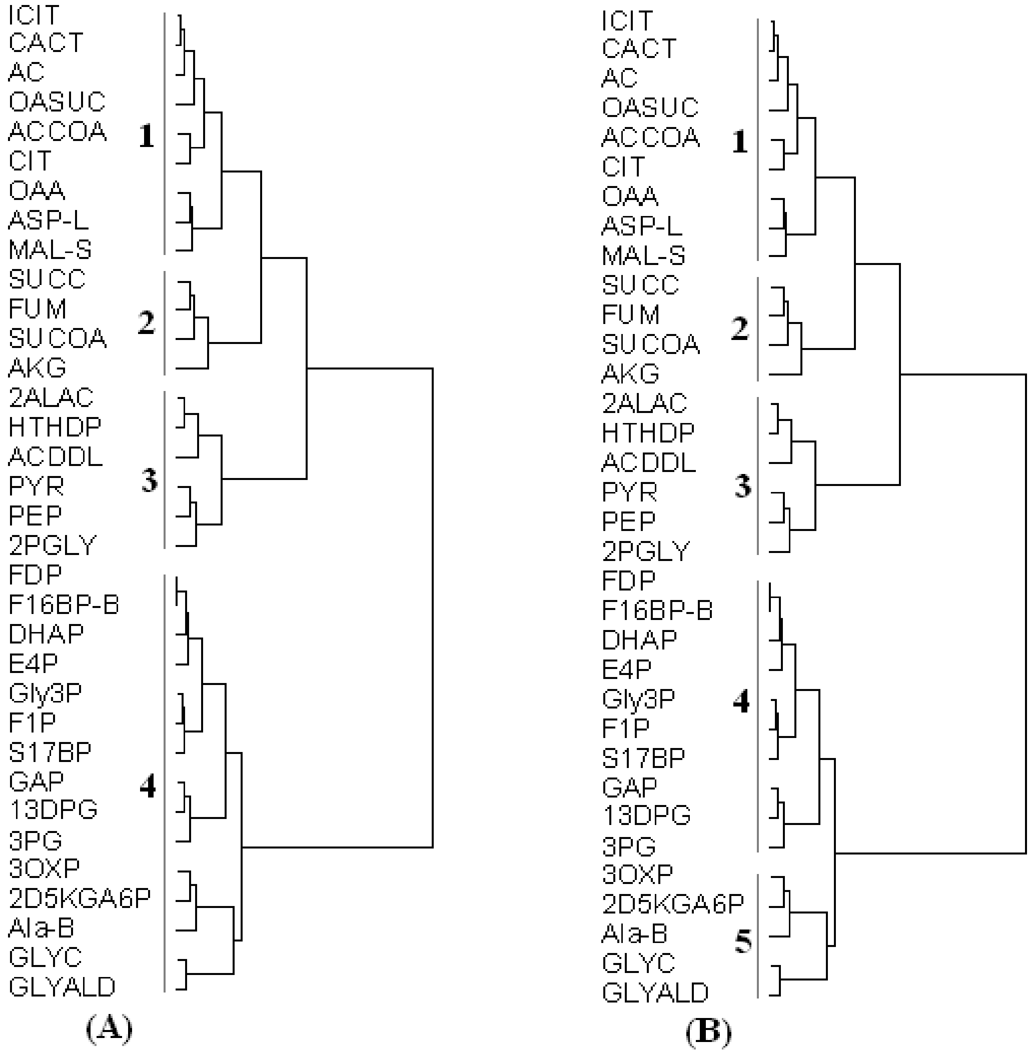

Figure S9 Dendrogram for the Core of the GSC of ape network

(A) The hierarchical tree is cut into 4 sub-trees (B) The hierarchical tree is cut into 5 sub-trees

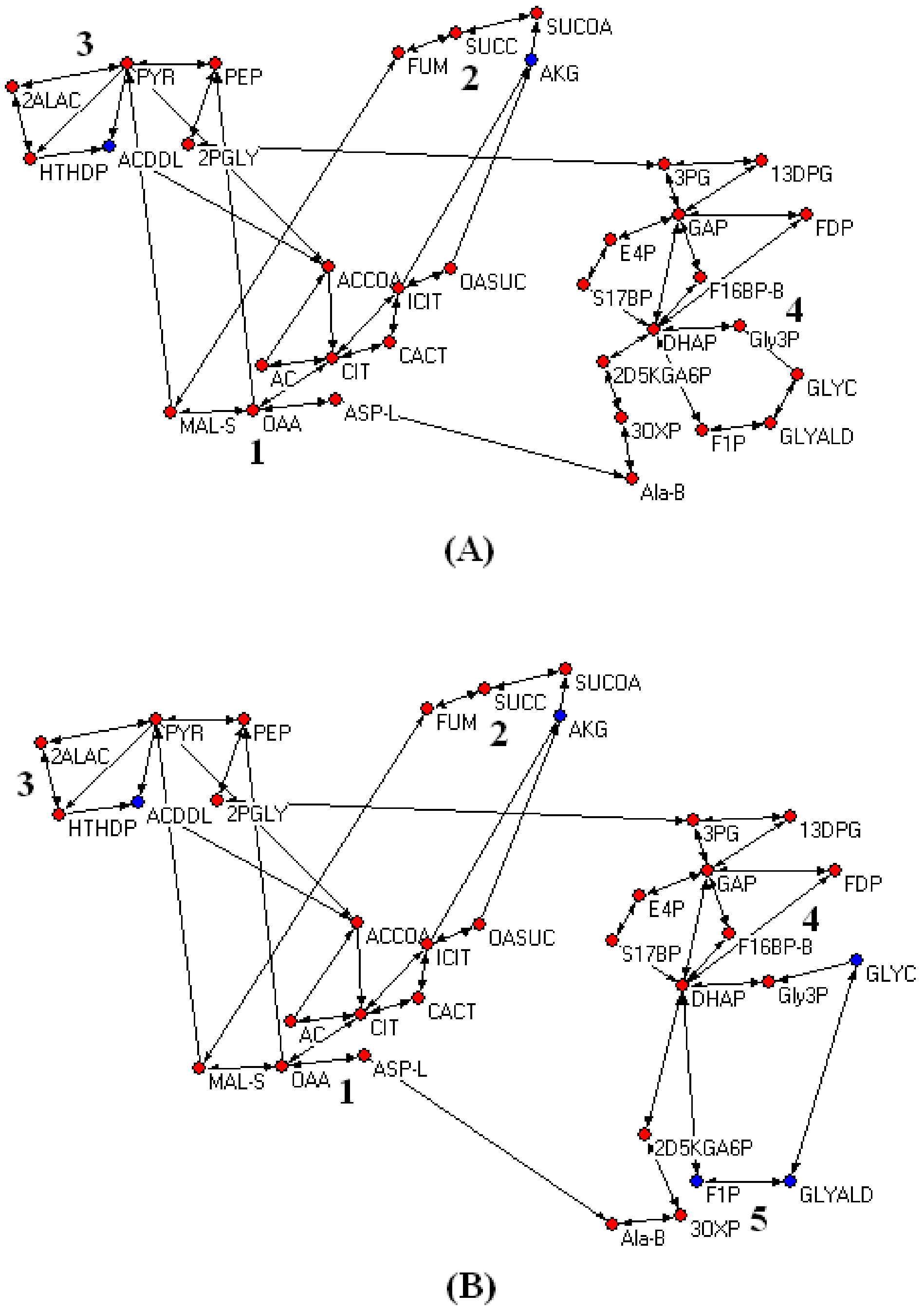

Figure.S10 Decomposition of the Core for the GSC of ape. The nodes included in the biggest strongly connected component of each cluster are shown in red colour.

(A) Decomposition corresponding to the sub-trees in FigS9 (A);

(B) Decomposition corresponding to the sub-trees in FigS9 (B)

3. Expand the clusters of the Core to the whole metabolic network by the "majority rule".

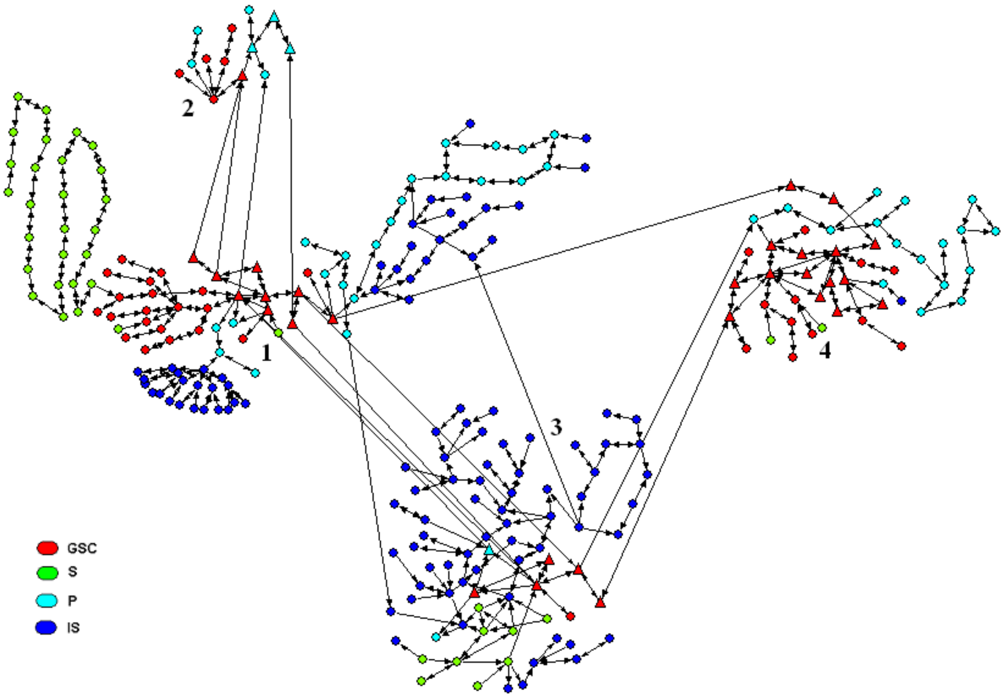

Figure S11 Decomposition of the ape metabolic network by expanding the clustering of the Core. Triangles correspond to the nodes of the Core. The four parts (GSC, S, P, IS) of bow tie structure are shown in distinct colours for the modules.

Table S1 Node distributions in the global structure of sub-networks obtained from the decomposition for ape network and all the unions which exhibit bow-tie structure

Cluster	Total nodes	GSC		S		P		IS		Bow-tie
		nodes	percent	nodes	percent	nodes	percent	nodes	percent	
1	120	29	24.17%	28	23.33%	22	18.33%	41	34.17%	Y
2	13	6	46.15%	0	0.00%	7	53.85%	0	0.00%	D
3	76	6	7.89%	9	11.84%	2	2.63%	59	77.63%	D
4	47	27	57.45%	2	4.26%	17	36.17%	1	2.13%	Y
1+2	133	39	29.32%	28	21.05%	25	18.80%	41	30.83%	Y
1+3	196	36	18.37%	40	20.41%	25	12.76%	95	48.47%	Y
3+4	123	33	26.83%	11	8.94%	19	15.45%	60	48.78%	Y
1+2+3	209	46	22.01%	39	18.66%	28	13.40%	96	45.93%	Y
1+3+4	243	63	25.93%	41	16.87%	42	17.28%	97	39.92%	Y
1+2+3+4	256	73	28.52%	41	16.02%	45	17.58%	97	37.89%	Y

Note: The 2nd and 3rd cluster can be conceived degraded bow-ties, because sub-network 2 does not have S part, while the GSC part of sub-network 3 is not the dominantly biggest strongly connected component, i.e., the second biggest strongly connected component has similar size as the GSC. Although sub-networks 2 and 3 are degraded bow-ties, combined sub-networks are all bow-tie structural networks.

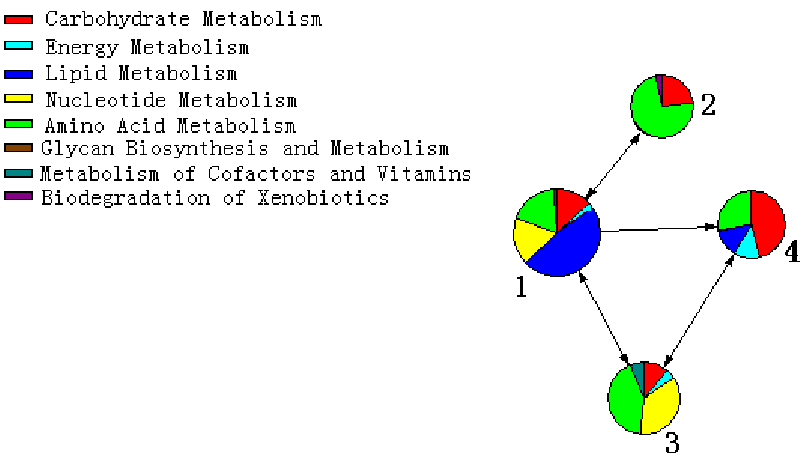

Figure.S12 Cartographic representation of the metabolic network for ape.

III. Decomposition result for the network of *Saccharomyces cerevisiae* iND750(sce)

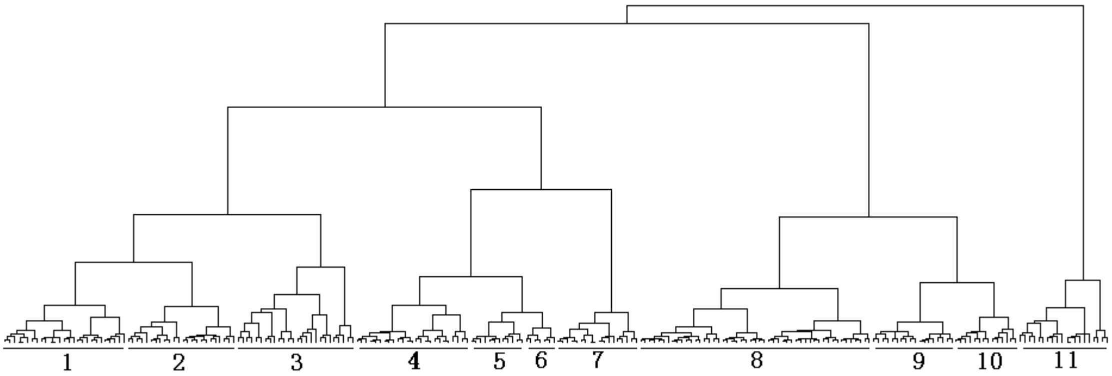

Figure S13 Dendrogram for the Core of the GSC of sce network

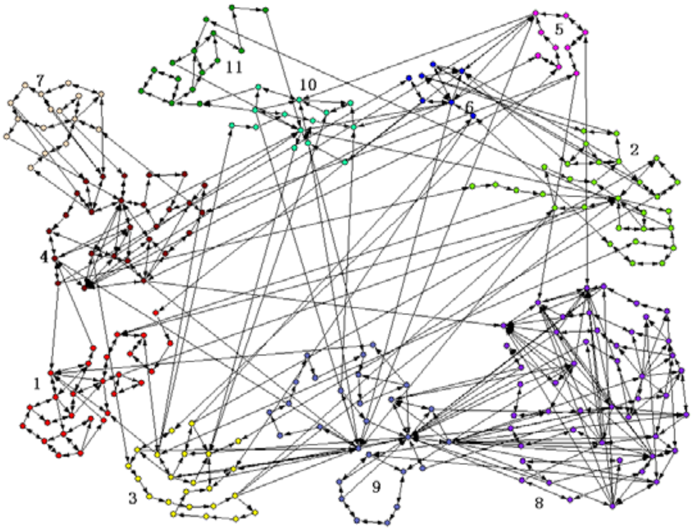

Figure S14 Decomposition of the Core for the GSC of sce

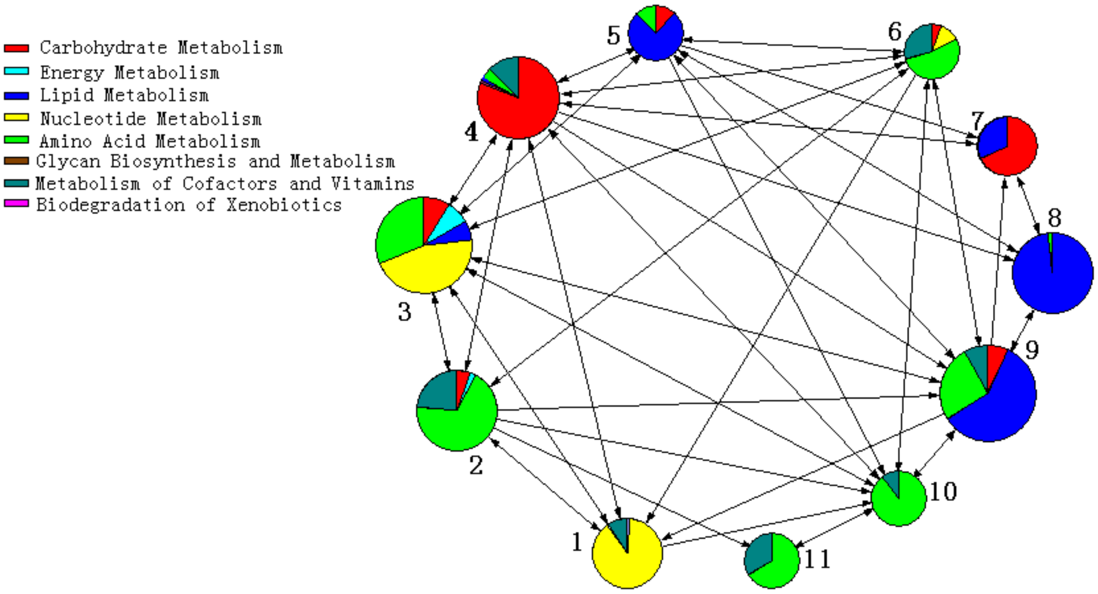

Figure S15 Cartographic representation of the metabolic network for sce.

Table S2 Node distributions in the global structure of sub-networks obtained from the decomposition for sce network

Cluster	Total nodes	GSC		S		P		IS		Bow-tie
		nodes	percent	nodes	percent	nodes	percent	nodes	percent	
1	51	21	41.18%	5	9.80%	8	15.69%	17	33.33%	Y
2	66	21	31.82%	11	16.67%	27	40.91%	7	10.61%	Y
3	90	25	27.78%	1	1.11%	59	65.56%	5	5.56%	Y
4	68	37	54.41%	5	7.35%	20	29.41%	6	8.82%	Y
5	19	5	26.31%	5	26.31%	7	36.84%	2	10.53%	Y
6	24	6	25.00%	0	0	18	75.00%	0	0	D

7	38	13	34.21%	5	13.16%	18	47.37%	2	5.26%	Y
8	65	26	40.00%	3	4.62%	24	36.92%	12	18.46%	Y
9	92	14	15.22%	24	26.09%	33	35.87%	21	22.83%	Y
10	22	5	22.73%	9	40.91%	7	31.82%	1	4.55%	Y
11	21	6	28.57%	1	4.76%	12	57.14%	2	9.52%	Y
Global network	556	269	48.38%	39	7.01%	229	41.19%	19	3.42%	Y

IV. Node distributions in the sub-networks of e.coli network obtained by different union of the twelve clusters

Table S3 Node distributions in 150 different sub-networks of *E.coli* network obtained by different union of the twelve clusters. For example, sub-network denoted by {1,0,1,1,1,0,0,1,0,0,0,0} is the union of the 1st, 3rd, 4th, 5th, and 8th cluster. The global network is denoted by {1,1,1,1,1,1,1,1,1,1,1,1}, which is presented here for comparison. The total absolute error is the sum of absolute errors between the percentage of the four parts of the sub-network and those of the global network. The average absolute error is 0.0854.

Number of clusters	Sub-network	Number of nodes	GSC Percentage	S Percentage	P Percentage	IS Percentage	Total absolute error
12	{1,1,1,1,1,1,1,1,1,1,1,1}	575	0.407	0.1478	0.3078	0.1883	0
11	{1,1,1,1,1,1,1,1,1,1,1,0}	558	0.4014	0.1505	0.3065	0.1912	0.0138
11	{1,1,1,1,1,1,1,1,1,0,1,1}	547	0.404	0.1554	0.2962	0.1774	0.0292
11	{1,0,1,1,1,1,1,1,1,1,1,1}	515	0.4078	0.1631	0.2932	0.1734	0.0322
11	{1,1,1,0,1,1,1,1,1,1,1,1}	531	0.3823	0.1638	0.3107	0.214	0.0493
11	{1,1,1,1,1,1,0,1,1,1,1,1}	526	0.4183	0.1217	0.3194	0.1875	0.0523
11	{0,1,1,1,1,1,1,1,1,1,1,1}	509	0.4028	0.1257	0.3183	0.1977	0.0526
11	{1,1,0,1,1,1,1,1,1,1,1,1}	552	0.3696	0.1612	0.3188	0.2324	0.0748
11	{1,1,1,1,1,1,1,1,0,1,1,1}	481	0.4179	0.1622	0.3222	0.205	0.0794
10	{1,0,1,1,1,1,0,1,1,1,1,1}	466	0.4206	0.1352	0.3047	0.1709	0.0314
10	{0,0,1,1,1,1,1,1,1,1,1,1}	449	0.4053	0.1403	0.3007	0.1775	0.0326
10	{1,1,1,1,1,1,0,1,1,0,1,1}	498	0.4157	0.1285	0.3072	0.1753	0.0398
10	{1,0,1,1,1,1,1,1,1,1,1,0}	498	0.4016	0.1667	0.2912	0.1761	0.0441
10	{1,1,1,1,1,1,1,1,1,0,1,0}	530	0.3981	0.1585	0.2943	0.1798	0.0448
10	{1,1,1,1,1,1,0,1,1,1,1,0}	509	0.4126	0.1238	0.3183	0.1906	0.0481
10	{0,1,1,1,1,1,1,1,1,0,1,1}	481	0.3992	0.1331	0.3056	0.1857	0.0495
10	{1,1,1,0,1,1,0,1,1,1,1,1}	482	0.3921	0.1369	0.3237	0.2158	0.0516
10	{1,1,1,0,1,1,1,1,1,0,1,1}	503	0.3877	0.163	0.2982	0.1934	0.0578
10	{0,1,1,1,1,1,1,1,1,1,1,0}	492	0.3963	0.128	0.3171	0.2012	0.0609
10	{1,0,1,1,1,1,1,1,1,0,1,1}	487	0.4045	0.1725	0.2793	0.1602	0.062
10	{1,1,1,0,1,1,1,1,1,1,1,0}	514	0.3755	0.1673	0.3093	0.2178	0.063

10	{1,1,0,1,1,1,1,1,0,1,1,1}	458	0.3886	0.1812	0.3079	0.2118	0.067
10	{1,1,1,1,1,1,1,1,0,0,1,1}	453	0.415	0.1722	0.3091	0.1928	0.0673
10	{1,1,1,1,1,1,1,1,0,1,1,0}	464	0.4116	0.1659	0.3211	0.2090	0.0721
10	{0,1,1,1,1,1,1,1,0,1,1,1}	415	0.4145	0.1373	0.3373	0.2191	0.0741
10	{0,1,1,0,1,1,1,1,1,1,1,1}	465	0.3742	0.1419	0.3226	0.2279	0.0774
10	{1,0,0,1,1,1,1,1,1,1,1,1}	471	0.38	0.1826	0.2951	0.201	0.0794
10	{1,1,0,1,1,1,0,1,1,1,1,1}	503	0.3777	0.1352	0.332	0.2359	0.0838
10	{1,0,0,1,1,1,1,1,1,1,1,1}	492	0.3659	0.1748	0.3049	0.2208	0.0881
10	{1,0,1,1,1,1,1,1,0,1,1,1}	421	0.4204	0.1829	0.3064	0.1892	0.097
10	{0,1,1,1,1,1,0,1,1,1,1,1}	460	0.4152	0.0935	0.3326	0.1978	0.1086
10	{1,1,1,0,1,1,1,1,0,1,1,1}	437	0.389	0.1831	0.3272	0.2379	0.1094
10	{1,1,1,1,1,1,0,1,0,1,1,1}	432	0.4329	0.1319	0.338	0.2060	0.1122
10	{0,1,0,1,1,1,1,1,1,1,1,1}	486	0.3601	0.1358	0.3313	0.2469	0.1178
9	{1,0,1,1,1,1,0,1,1,1,1,0}	449	0.4143	0.1381	0.3029	0.1737	0.0293
9	{1,0,1,0,1,1,0,1,1,1,1,1}	422	0.391	0.154	0.3081	0.2014	0.032
9	{1,1,0,1,1,1,0,1,0,1,1,1}	409	0.401	0.1516	0.3227	0.2134	0.0374
9	{1,1,1,1,1,1,0,1,1,0,1,0}	481	0.4096	0.131	0.3056	0.1780	0.038
9	{1,1,1,1,1,1,1,1,1,0,0,0}	512	0.3906	0.1543	0.3027	0.1946	0.0429
9	{1,1,1,0,1,1,0,1,1,0,1,1}	454	0.3987	0.1344	0.3106	0.1931	0.0435
9	{1,0,1,1,1,1,0,1,1,0,1,1}	438	0.4178	0.1438	0.29	0.1560	0.0436
9	{0,0,1,1,1,1,1,1,1,1,1,0}	432	0.3981	0.1435	0.2986	0.1805	0.0447
9	{1,1,1,1,1,0,0,1,1,0,1,1}	477	0.4067	0.1216	0.3208	0.1971	0.053
9	{1,1,1,0,1,1,0,1,1,1,1,0}	465	0.3849	0.1398	0.3226	0.2201	0.0602
9	{1,1,1,1,1,1,1,1,0,0,1,0}	436	0.4083	0.1766	0.3073	0.1964	0.0602
9	{1,1,1,1,1,1,1,0,1,0,1,0}	511	0.3992	0.1644	0.2838	0.1670	0.0636
9	{0,0,1,1,1,1,1,1,0,1,1,1}	355	0.4197	0.1577	0.3183	0.1971	0.0663
9	{1,0,0,1,1,1,0,1,1,1,1,1}	443	0.3747	0.1467	0.3183	0.2235	0.0668
9	{1,1,1,1,1,0,0,1,1,1,1,0}	488	0.4037	0.1168	0.332	0.2124	0.0686
9	{0,0,1,0,1,1,1,1,1,1,1,1}	405	0.3728	0.1605	0.3037	0.2099	0.0766
9	{1,1,1,1,1,1,0,1,0,0,1,1}	404	0.4307	0.1411	0.3243	0.1923	0.0803
9	{1,0,1,1,1,1,1,1,1,0,1,0}	470	0.3979	0.1766	0.2766	0.1624	0.0806
9	{1,1,1,0,1,1,1,1,0,0,1,1}	409	0.3961	0.1834	0.313	0.2144	0.0815
9	{1,1,1,0,1,1,0,1,0,1,1,1}	388	0.4021	0.1521	0.3454	0.2431	0.0837
9	{0,0,1,1,1,1,0,1,1,1,1,1}	400	0.42	0.105	0.315	0.175	0.0856
9	{1,0,1,1,1,1,1,1,0,1,1,0}	404	0.4134	0.1881	0.3045	0.1931	0.0933
9	{1,0,1,1,1,1,0,1,0,1,1,1}	372	0.4382	0.1505	0.3226	0.1881	0.0974
9	{1,0,1,0,1,1,1,1,1,0,1,1}	443	0.386	0.1828	0.2799	0.1768	0.0977
9	{1,1,1,1,1,1,0,1,0,1,1,0}	415	0.4265	0.1349	0.3373	0.2103	0.0981
9	{1,0,1,0,1,1,1,1,1,1,1,0}	454	0.3722	0.1872	0.293	0.2049	0.0992
9	{0,0,0,1,1,1,1,1,1,1,1,1}	426	0.3568	0.1526	0.3146	0.2324	0.1005
9	{1,0,0,1,1,1,1,1,0,1,1,1}	398	0.3869	0.206	0.2889	0.1959	0.1165
9	{1,0,1,1,1,1,1,1,0,0,1,1}	393	0.4173	0.1959	0.2901	0.1739	0.1168
9	{1,1,1,0,1,1,1,1,0,1,1,0}	420	0.381	0.1881	0.3262	0.2436	0.1173

9	{1,1,0,0,1,1,1,1,0,1,1,1}	414	0.3696	0.1908	0.3261	0.252	0.1226
9	{1,1,1,1,1,0,0,1,0,1,1,1}	411	0.4234	0.1241	0.3552	0.2327	0.1276
9	{1,0,1,0,1,1,1,1,0,1,1,1}	377	0.3873	0.2095	0.3103	0.2253	0.1285
9	{0,0,1,1,1,1,1,1,1,0,1,1}	421	0.3444	0.1425	0.342	0.2739	0.1357
8	{1,0,0,1,1,1,0,1,0,1,1,1}	349	0.4011	0.1748	0.3037	0.1958	0.0541
8	{1,0,1,0,1,0,0,1,1,1,1,1}	401	0.3791	0.1471	0.3242	0.2285	0.0572
8	{1,1,1,0,1,1,0,1,0,0,1,1}	360	0.4111	0.15	0.3306	0.2167	0.0582
8	{0,0,0,1,1,1,1,1,0,1,1,1}	332	0.3795	0.1837	0.2982	0.2058	0.0742
8	{0,1,0,1,1,1,0,1,0,1,1,1}	343	0.3936	0.1195	0.3411	0.2322	0.0834
8	{1,0,1,1,1,1,0,1,0,0,1,1}	344	0.436	0.1628	0.3052	0.1705	0.0881
8	{0,0,1,0,1,1,0,1,1,1,1,1}	356	0.3848	0.1236	0.3202	0.2116	0.0927
8	{1,1,1,1,1,0,0,1,0,0,1,1}	383	0.4204	0.1332	0.342	0.2201	0.0952
8	{1,1,0,0,1,1,0,1,0,1,1,1}	365	0.3808	0.1589	0.3452	0.2593	0.097
8	{1,0,0,1,1,0,0,1,1,1,1,1}	422	0.3626	0.1398	0.3341	0.2503	0.1048
8	{0,0,1,1,1,0,0,1,1,1,1,1}	379	0.409	0.095	0.3325	0.2023	0.1057
8	{1,0,1,1,1,0,0,1,0,1,1,1}	351	0.4274	0.1425	0.3419	0.2184	0.1089
8	{0,1,0,0,1,1,1,1,0,1,1,1}	348	0.3563	0.1667	0.3448	0.2777	0.1118
8	{0,1,1,1,1,1,0,1,0,0,1,1}	338	0.429	0.1065	0.3432	0.2071	0.1148
8	{1,0,1,0,1,1,1,1,0,0,1,1}	349	0.3954	0.212	0.2923	0.1968	0.1284
7	{0,0,0,1,1,1,0,1,0,1,1,1}	283	0.3958	0.1413	0.318	0.2072	0.0354
7	{1,0,1,0,1,0,0,1,1,0,1,1}	373	0.3861	0.1448	0.3083	0.2019	0.0479
7	{1,0,0,1,1,0,0,1,0,1,1,1}	328	0.3872	0.1677	0.3232	0.2287	0.0705
7	{1,0,1,1,1,0,0,1,0,0,1,1}	323	0.4241	0.1548	0.3251	0.2023	0.0828
7	{0,0,1,0,1,1,0,1,0,1,1,1}	262	0.3969	0.1412	0.3511	0.2506	0.0867
7	{0,1,1,0,1,1,0,1,0,0,1,1}	294	0.4048	0.1122	0.3537	0.2391	0.0918
7	{0,0,1,0,1,1,0,1,1,0,1,1}	328	0.3933	0.1189	0.3018	0.1798	0.0972
7	{1,0,0,0,1,1,0,1,0,1,1,1}	305	0.377	0.1869	0.3279	0.2481	0.1184
7	{1,1,0,0,1,0,0,1,0,1,1,1}	344	0.3663	0.1512	0.3663	0.2946	0.1237
7	{0,1,0,0,1,1,0,1,0,1,1,1}	299	0.3679	0.1237	0.3712	0.2909	0.1269
7	{0,1,0,1,1,0,0,1,0,1,1,1}	322	0.3789	0.1087	0.3634	0.2681	0.1345
7	{0,1,1,1,1,0,0,1,0,0,1,1}	317	0.4164	0.0946	0.3659	0.2418	0.1351
6	{1,1,1,1,1,1,0,0,0,0,0,0}	350	0.4171	0.1457	0.3229	0.2010	0.0504
6	{1,1,0,1,1,1,0,1,0,0,0,0}	346	0.3815	0.1561	0.3179	0.2215	0.051
6	{1,0,1,0,1,0,0,1,0,0,1,1}	279	0.3978	0.1685	0.3333	0.2353	0.0924
6	{0,0,0,0,1,1,0,1,0,1,1,1}	239	0.364	0.1506	0.3515	0.2762	0.093
6	{0,0,0,1,1,0,0,1,0,1,1,1}	262	0.3779	0.1298	0.3435	0.2493	0.0943
6	{1,1,1,1,1,0,0,1,0,0,0,0}	348	0.4023	0.1293	0.3563	0.2499	0.097
6	{1,1,0,1,1,1,0,0,1,0,0,0}	421	0.3587	0.1425	0.3183	0.2327	0.1072
6	{1,1,0,0,1,1,1,1,0,0,0,0}	351	0.359	0.188	0.3219	0.2525	0.1086
6	{1,1,0,1,1,1,1,0,0,0,0,0}	376	0.3697	0.1995	0.2872	0.2029	0.1158
6	{0,0,1,0,1,0,0,1,1,0,1,1}	307	0.3779	0.1075	0.3225	0.2139	0.1389
5	{1,1,0,1,1,1,0,0,0,0,0,0}	327	0.3823	0.1651	0.3028	0.2039	0.0594
5	{1,0,1,1,1,0,0,0,1,0,0,0}	363	0.3912	0.1405	0.3003	0.1864	0.0612

5	{1,1,1,0,1,1,0,0,0,0,0,0}	306	0.3922	0.1569	0.3301	0.2309	0.0627
5	{1,0,1,1,1,0,0,0,0,0,1,0}	287	0.4181	0.1707	0.3066	0.1869	0.0681
5	{0,1,1,0,1,1,0,0,0,0,1,0}	258	0.3953	0.124	0.3372	0.2274	0.0709
5	{1,1,1,1,1,0,0,0,0,0,0,0}	329	0.4043	0.1368	0.3435	0.2340	0.0713
5	{1,1,1,0,1,0,0,0,0,0,1,0}	303	0.3894	0.1551	0.3366	0.2409	0.0723
5	{1,0,1,1,1,0,0,1,0,0,0,0}	288	0.4028	0.1528	0.3403	0.2361	0.0749
5	{1,1,0,0,1,1,0,1,0,0,0,0}	302	0.3709	0.149	0.3444	0.2616	0.0755
5	{1,0,1,1,1,1,0,0,0,0,0,0}	290	0.4207	0.1724	0.3	0.177	0.0766
5	{1,1,0,1,1,0,0,1,0,0,0,0}	325	0.3662	0.1477	0.3385	0.2564	0.0819
5	{0,1,1,1,1,1,0,0,0,0,0,0}	284	0.412	0.1056	0.3451	0.2206	0.0846
5	{1,0,0,1,1,1,0,1,0,0,0,0}	286	0.3776	0.1853	0.2937	0.2016	0.087
5	{0,1,1,0,1,1,1,0,0,0,0,0}	289	0.3633	0.1661	0.3287	0.2514	0.0874
5	{1,0,1,0,1,1,0,1,0,0,0,0}	265	0.3887	0.1774	0.3245	0.2326	0.0926
5	{1,1,1,1,0,0,0,0,1,0,0,0}	287	0.4077	0.1533	0.2613	0.1277	0.093
5	{0,1,1,1,1,0,0,0,0,0,1,0}	281	0.4093	0.1032	0.3523	0.2312	0.0936
5	{1,0,1,0,1,0,0,0,1,0,1,0}	337	0.3769	0.1573	0.2908	0.1889	0.0943
5	{1,0,1,0,1,0,0,1,1,0,0,0}	338	0.3639	0.142	0.3195	0.2307	0.0978
5	{1,0,1,0,1,1,0,0,0,0,1,0}	264	0.4053	0.197	0.2879	0.1793	0.0984
5	{1,0,1,0,1,1,0,0,1,0,0,0}	340	0.3794	0.1588	0.2853	0.1804	0.1002
5	{1,1,0,0,1,1,1,0,0,0,0,0}	332	0.3584	0.1988	0.3072	0.2369	0.1021
5	{1,0,1,0,1,0,0,1,0,0,1,0}	262	0.3855	0.1756	0.3321	0.2443	0.1041
5	{1,1,1,0,0,0,0,0,1,0,1,0}	261	0.41	0.1571	0.2529	0.1162	0.1099
5	{1,1,1,0,1,0,0,0,1,0,0,0}	379	0.3694	0.1293	0.3245	0.2295	0.1122
5	{0,1,0,1,1,1,1,0,0,0,0,0}	310	0.3548	0.1742	0.3	0.2215	0.12
5	{1,1,1,0,1,0,0,1,0,0,0,0}	304	0.375	0.1382	0.3684	0.2872	0.1212
5	{1,1,0,1,1,0,0,0,0,0,1,0}	324	0.3457	0.1481	0.3056	0.2263	0.127
5	{1,0,0,1,1,1,0,0,1,0,0,0}	361	0.3518	0.1579	0.2992	0.2170	0.1276
4	{1,1,0,0,1,1,0,0,0,0,0,0}	283	0.371	0.159	0.3286	0.2438	0.0719
4	{1,1,0,1,1,0,0,0,0,0,0,0}	306	0.366	0.1569	0.3235	0.2396	0.082
4	{1,0,1,0,1,0,0,1,0,0,0,0}	246	0.3902	0.1911	0.3049	0.2101	0.0866
4	{1,0,1,0,1,0,0,0,0,0,1,0}	243	0.3868	0.1893	0.3128	0.2223	0.093
4	{1,1,1,0,1,0,0,0,0,0,0,0}	285	0.3754	0.1474	0.3544	0.2714	0.0932
4	{1,0,1,0,1,0,0,0,1,0,0,0}	319	0.3636	0.1505	0.3041	0.2132	0.0942
4	{1,1,1,0,0,0,0,0,1,0,0,0}	243	0.3951	0.1481	0.2675	0.1426	0.1044
4	{1,0,0,1,1,1,0,0,0,0,0,0}	267	0.3783	0.1985	0.2734	0.1785	0.1262
4	{1,0,1,0,1,0,0,1,0,0,0,0}	244	0.3689	0.168	0.3525	0.2800	0.1297
3	{1,1,0,0,1,0,0,0,0,0,0,0}	262	0.3511	0.1489	0.355	0.2889	0.1117
3	{1,0,1,0,1,0,0,0,0,0,0,0}	225	0.3689	0.1822	0.3333	0.2592	0.1199
3	{1,0,0,1,1,0,0,0,0,0,0,0}	246	0.3577	0.1911	0.2967	0.2208	0.1207
3	{1,1,0,1,0,0,0,0,0,0,0,0}	170	0.3941	0.2118	0.2706	0.1686	0.1279
3	{1,1,1,0,0,0,0,0,0,0,0,0}	149	0.4497	0.1678	0.3154	0.1766	0.1406
2	{0,1,0,1,0,0,0,0,0,0,0,0}	104	0.3654	0.1442	0.2981	0.2019	0.1098
2	{1,0,0,0,1,0,0,0,0,0,0,0}	202	0.3366	0.1881	0.3317	0.2805	0.1407

2	{0,0,0,0,1,1,0,0,0,0,0,0}	157	0.3376	0.1465	0.3248	0.2568	0.1414

V. Randomized networks of E.coli network

Table S4 Topological metrics of 60 randomized network of E.coli network

C: Average clustering coefficient of the network

M: Modularity metric of the network obtained by simulated annealing algorithm

Network	GSC	S	P	IS	Core	C	M
1	296	111	107	61	212	0.0002	0.7619
2	270	86	138	81	189	0.0007	0.7532
3	300	90	133	52	221	0.0036	0.7540
4	237	116	105	117	161	0.0092	0.7652
5	313	75	134	64	220	0.0052	0.7642
6	305	84	122	64	223	0.0034	0.7580
7	284	85	150	56	202	0.004	0.7568
8	304	94	132	45	219	0.0011	0.7587
9	296	88	120	71	213	0.0027	0.7634
10	291	81	151	52	212	0.0016	0.7603
11	291	71	118	95	199	0.0005	0.7544
12	297	81	129	68	205	0.0031	0.7610
13	295	100	126	54	209	0.0007	0.7616
14	300	71	133	71	205	0.0061	0.7574
15	310	92	118	55	220	0.0022	0.7583
16	289	86	132	68	207	0.0019	0.7638
17	283	99	120	73	203	0.003	0.7612
18	282	89	126	78	192	0.0063	0.7542
19	289	93	108	85	203	0.0014	0.7567
20	297	85	114	79	202	0.0024	0.7601
21	275	97	130	73	195	0.0018	0.7592
22	293	74	125	83	204	0.0023	0.7600
23	311	84	134	46	223	0.0039	0.7593
24	271	83	132	89	194	0.0012	0.7599
25	259	89	147	80	187	0.002	0.7638
26	272	94	146	63	195	0.0018	0.7571
27	300	89	110	76	219	0.0031	0.7630
28	274	99	110	92	197	0.0062	0.7654
29	307	78	122	68	218	0.0019	0.7522
30	264	108	145	58	190	0.0014	0.7513
31	276	107	127	65	209	0.0041	0.7558
32	291	86	125	73	207	0.0021	0.7634
33	284	97	136	58	207	0.0046	0.7553
34	297	90	120	68	212	0.0027	0.7605

35	296	78	112	89	211	0.0026	0.7622
36	313	84	115	63	227	0.0029	0.7675
37	291	86	123	75	202	0.0031	0.7698
38	270	102	128	75	198	0.0009	0.7639
39	290	105	112	68	208	0.0009	0.7648
40	279	113	102	81	207	0.0035	0.7652
41	278	86	137	74	200	0	0.7570
42	289	89	112	85	198	0.0035	0.7554
43	286	81	137	71	200	0.0019	0.7653
44	270	88	164	53	185	0.0022	0.7614
45	303	79	102	91	205	0.004	0.7556
46	254	93	155	73	187	0.0076	0.7645
47	296	102	124	53	205	0.0007	0.7560
48	284	88	109	94	204	0.0006	0.7634
49	273	106	121	75	199	0.0049	0.7609
50	307	87	102	79	226	0.0008	0.7620
51	302	84	134	55	227	0.0049	0.7611
52	260	107	121	87	194	0.0048	0.7597
53	294	85	125	71	214	0.0014	0.7636
54	284	92	142	57	197	0.0008	0.7649
55	286	97	120	72	206	0.0018	0.7551
56	266	80	158	71	183	0.0059	0.7552
57	305	83	131	56	219	0.0005	0.7552
58	310	94	107	64	216	0.0016	0.7659
59	293	89	132	61	194	0.0027	0.7649
60	290	90	130	65	210	0.0012	0.7544
**E.coli**	**234**	**85**	**177**	**79**	**163**	**0.0646**	**0.8527**

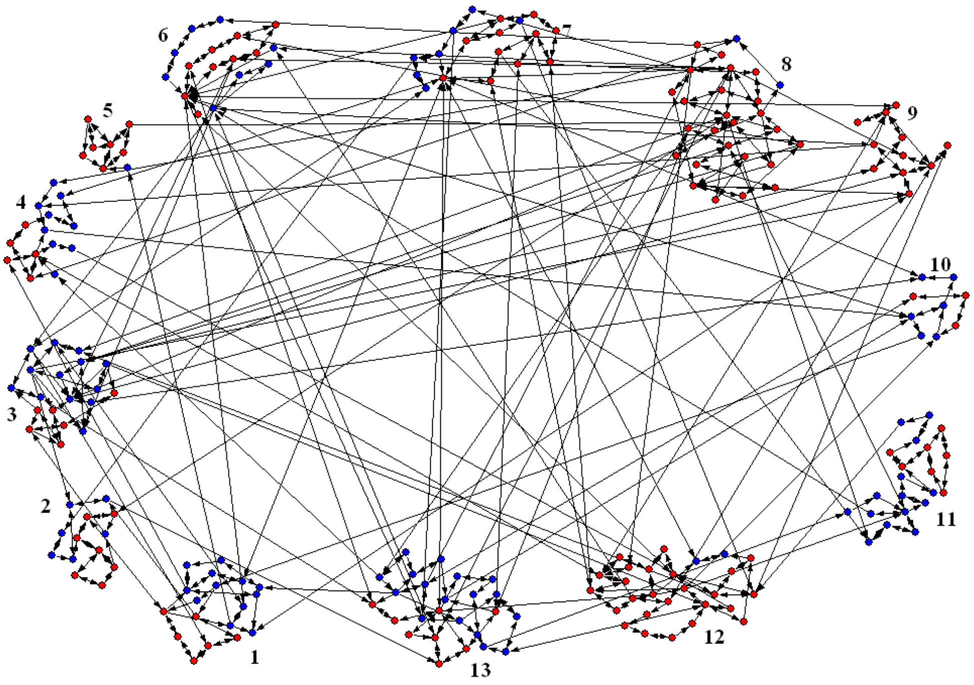

Figure S16 Decomposition of the Core for the 51st random network by simulated annealing algorithm. The modularity metric corresponding to this decomposition is 0.6538 (Because the Core is the most densely connected part of a network, its modularity metric is lower than that of the global network, which equals to 0.7611). The nodes included in the biggest strongly connected component of each cluster are shown in red color. Comparison with Figure 2 in the paper shows that the clusters here exhibit much lower extent of structural equivalence as equation (1) defines. That is to say, although this decomposition has relatively high modularity metric, it cannot lead to the result that each module has bow-tie structure.

VI. Discussion about the graph representation of metabolic networks and the decomposition algorithm

The metabolic network could be represented as different kinds of graph models as Figure S17 shows (also see Ref.[7] of the paper for a review). In this work, a metabolic network is represented by a simple directed graph whose nodes correspond to metabolites and arcs correspond to reactions between these metabolites, as shown in Figure S17(A). Since such representation is simple, and thus algorithms based on it are easier to design, it has widely been applied to describe metabolic networks. However, this graph model may raise a problem for reactions with more than 2 metabolites, such as $A+B \to C$. This reaction is converted to two arcs A → B and A → C in the network, and these two arcs should always be coupled from biological point of view. But the two coupled arcs may become un-coupled in the clustering process and random rewiring. In principle, they could be placed in different modules, leading to the aftereffect that this reaction is impossible to happen within one module.

To evaluate the probable influence of this flaw to our result, we checked our data carefully. The *E.coli* metabolic network includes 976 reactions, while only 145 of them have more than one substrate and/or product, but 49 ones are “un-coupled” into different modules. A simulation is also performed to evaluate the consequence of “un-coupling”. Only one substrate-product pair of

each reaction from those 145 is incorporated into the rest to form a pseudo E.coli network free of "coupling". The results of following decomposition and rewiring are summarized into Figure S18 and Table S5. Figure S18 has similar features as Figure 9 in the paper, while the result in Table S5 are similar those in Table 2 of the paper. The results indicate that, like E.coli network, the non-coupled pseudo E.coli network still exhibits modularity feature of bow-tie modules compared with the randomized version. Therefore, un-coupling is not likely to affect the conclusion of this paper.

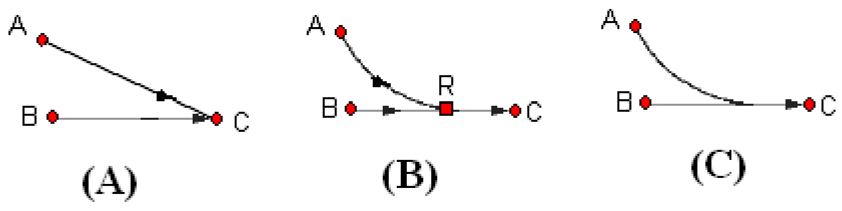

Figure S17 Different graph representations of a reaction $A + B \rightarrow C$
(A) simple directed graph (B) substrate-reaction bipartite graph (C) hypergraph

TableS5 Graph metrics of pseudo E.coli network and its randomized version

	GSC	S	P	IS	Core	C	M
(A) Pseudo E.coli network	149	76	152	63	89	0.0570	0.8651
(B) randomized version of Pseudo E.coli network	206	42	133	59	140	0.0036	0.7985

C: Average clustering coefficient of the network

M: Modularity metric of the network obtained by simulated annealing algorithm

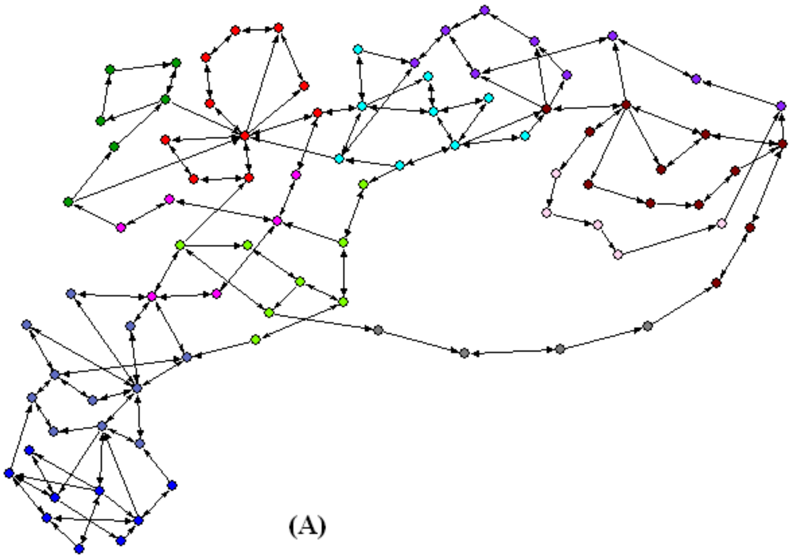

(A)

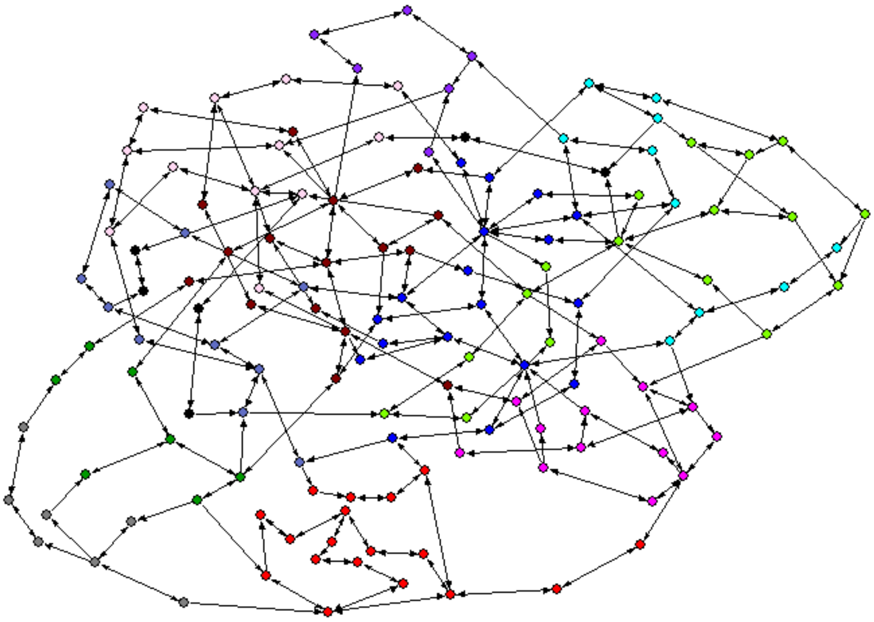

(B)

Figure S18 – Comparison of the Core of pseudo E.coli network with that of a randomized network.

(A) 11 clusters of the Core for pseudo E.coli network

(B) 11 clusters of the Core for a randomized network

Both of the Cores are decomposed by our algorithm. Different clusters are shown in different colours. These two networks include 89 and 140 nodes respectively.

VII. Node distribution in the sub-networks of carbohydrate metabolism

Table S6 Node distribution in the sub-networks of carbohydrate metabolism for the 8 eukaryote organisms in the database of Ma and Zeng

Organism	Total Nodes	GSC		S		P		IS		Bow-tie
		nodes	percent	nodes	percent	nodes	percent	nodes	percent	
hsa	91	40	43.96%	17	18.68%	13	14.29%	21	23.08%	Y
mmu	60	24	40.00%	7	11.67%	5	8.33%	24	40.00%	Y
rno	40	17	42.50%	7	17.50%	9	22.50%	7	17.50%	Y
dme	75	36	48.00%	15	20.00%	15	20.00%	9	12.00%	Y
cel	71	41	57.75%	10	14.08%	14	19.72%	6	8.45%	Y
ath	91	47	51.65%	26	28.57%	10	10.99%	8	8.79%	Y
sce	86	52	60.47%	18	20.93%	9	10.47%	7	8.14%	Y
spo	76	28	36.84%	11	14.47%	28	36.84%	9	11.84%	Y

Table S7 Node distribution in the sub-networks of carbohydrate metabolism for the 56 bacteria organisms in the database of Ma and Zeng

Organism	Total nodes	GSC		S		P		IS		Bow-tie
		nodes	percent	nodes	percent	nodes	percent	nodes	percent	
eco	164	76	46.34%	45	27.44%	20	12.20%	23	14.02%	Y
ecc	128	73	57.03%	24	18.75%	15	11.72%	16	12.50%	Y
sty	151	71	47.02%	37	24.50%	20	13.25%	23	15.23%	Y
stm	157	72	45.86%	40	25.48%	20	12.74%	25	15.92%	Y
ype	134	55	41.04%	37	27.61%	11	8.21%	31	23.13%	Y
ypk	128	52	40.63%	32	25.00%	12	9.38%	32	25.00%	Y
sfl	140	67	47.86%	35	25.00%	12	8.57%	26	18.57%	Y
hin	66	43	65.15%	14	21.21%	6	9.09%	3	4.55%	Y
pmu	71	46	64.79%	11	15.49%	10	14.08%	4	5.63%	Y
xfa	52	52	100.00%	0	0.00%	0	0.00%	0	0.00%	D
xcc	93	57	61.29%	14	15.05%	15	16.13%	7	7.53%	Y
xac	97	59	60.82%	16	16.49%	14	14.43%	8	8.25%	Y
vch	90	57	63.33%	12	13.33%	11	12.22%	10	11.11%	Y
pae	74	49	66.22%	15	20.27%	5	6.76%	5	6.76%	Y
son	78	50	64.10%	5	6.41%	18	23.08%	5	6.41%	Y
nme	74	50	67.57%	7	9.46%	16	21.62%	1	1.35%	Y
rso	91	51	56.04%	23	25.27%	5	5.49%	12	13.19%	Y
cje	40	37	92.50%	1	2.50%	1	2.50%	1	2.50%	Y
mlo	113	47	41.59%	24	21.24%	22	19.47%	20	17.70%	Y
sme	128	49	38.28%	32	25.00%	29	22.66%	18	14.06%	Y
atu	122	58	47.54%	22	18.03%	24	19.67%	18	14.75%	Y
bme	97	55	56.70%	19	19.59%	17	17.53%	6	6.19%	Y
bms	84	40	47.62%	18	21.43%	20	23.81%	6	7.14%	Y

ccr	78	49	62.82%	17	21.79%	11	14.10%	1	1.28%	Y
bsu	111	52	46.85%	28	25.23%	17	15.32%	14	12.61%	Y
bha	105	49	46.67%	29	27.62%	13	12.38%	14	13.33%	Y
oih	96	54	56.25%	26	27.08%	15	15.63%	1	1.04%	Y
sav	70	50	71.43%	14	20.00%	4	5.71%	2	2.86%	Y
sam	70	50	71.43%	14	20.00%	4	5.71%	2	2.86%	Y
lmo	87	51	58.62%	13	14.94%	22	25.29%	1	1.15%	Y
lin	77	44	57.14%	17	22.08%	12	15.58%	4	5.19%	Y
lla	78	44	56.41%	16	20.51%	13	16.67%	5	6.41%	Y
spy	66	38	57.58%	18	27.27%	8	12.12%	2	3.03%	Y
spn	79	46	58.23%	14	17.72%	5	6.33%	14	17.72%	Y
san	68	39	57.35%	16	23.53%	11	16.18%	2	2.94%	Y
smu	84	49	58.33%	11	13.10%	11	13.10%	13	15.48%	Y
cac	83	45	54.22%	18	21.69%	13	15.66%	7	8.43%	Y
tte	63	32	50.79%	13	20.63%	9	14.29%	9	14.29%	Y
mpu	34	23	67.65%	4	11.76%	5	14.71%	2	5.88%	Y
uur	12	12	100.00%	0	0.00%	0	0.00%	0	0.00%	D
mtu	90	46	51.11%	13	14.44%	23	25.56%	8	8.89%	Y
mle	59	41	69.49%	3	5.08%	3	5.08%	12	20.34%	Y
cgl	81	47	58.02%	13	16.05%	16	19.75%	5	6.17%	Y
sco	110	49	44.55%	20	18.18%	24	21.82%	17	15.45%	Y
fnu	53	32	60.38%	3	5.66%	16	30.19%	2	3.77%	Y
ctr	38	19	50.00%	8	21.05%	9	23.68%	2	5.26%	Y
bbu	31	19	61.29%	0	0.00%	12	38.71%	0	0.00%	D
tpa	40	25	62.50%	5	12.50%	10	25.00%	0	0.00%	Y
lil	86	41	47.67%	12	13.95%	17	19.77%	16	18.60%	Y
syn	74	40	54.05%	11	14.86%	23	31.08%	0	0.00%	Y
tel	68	31	45.59%	15	22.06%	21	30.88%	1	1.47%	Y
ana	88	39	44.32%	14	15.91%	27	30.68%	8	9.09%	Y
cte	49	32	65.31%	7	14.29%	7	14.29%	3	6.12%	Y
dra	68	45	66.18%	12	17.65%	10	14.71%	1	1.47%	Y
aae	43	33	76.74%	1	2.33%	8	18.60%	1	2.33%	Y
tma	103	36	34.95%	29	28.16%	15	14.56%	23	22.33%	Y

Table S8 Node distribution in the sub-networks of carbohydrate metabolism for the 16 archaea organisms in the database of Ma and Zeng

Organism	Total nodes	GSC		S		P		IS		Bow-tie
		nodes	percent	nodes	percent	nodes	percent	nodes	percent	
mja	29	16	55.17%	2	6.90%	6	20.69%	5	17.24%	y
mac	38	23	60.53%	1	2.63%	13	34.21%	1	2.63%	y
mma	39	22	56.41%	1	2.56%	13	33.33%	3	7.69%	y
afu	31	19	61.29%	5	16.13%	3	9.68%	4	12.90%	y

hal	29	25	86.21%	3	10.34%	1	3.45%	0	0.00%	y
tac	34	28	82.35%	5	14.71%	1	2.94%	0	0.00%	y
tvo	35	30	85.71%	4	11.43%	1	2.86%	0	0.00%	y
ape	40	34	85.00%	5	12.50%	1	2.50%	0	0.00%	y
sso	41	27	65.85%	9	21.95%	1	2.44%	4	9.76%	y
sto	34	23	67.65%	6	17.65%	1	2.94%	4	11.76%	y
pai	38	30	78.95%	7	18.42%	1	2.63%	0	0.00%	y
